# Mixture distributions in a stochastic gene expression model with delayed feedback: a WKB approximation approach

**DOI:** 10.1007/s00285-020-01512-y

**Published:** 2020-06-24

**Authors:** Pavol Bokes, Alessandro Borri, Pasquale Palumbo, Abhyudai Singh

**Affiliations:** 1 Comenius University, Bratislava, Slovakia; 2 Mathematical Institute, Slovak Academy of Sciences, Bratislava, Slovakia; 3 Italian National Research Council (IASI-CNR), Rome, Italy; 4 University of Milano-Bicocca, Milan, Italy; 5 University of Delaware, Newark, DE USA

**Keywords:** Stochastic gene expression, Bursting, Production delay, WKB approximation, Large deviations, Exponential asymptotics, 92C40, 60J27, 41A60

## Abstract

Noise in gene expression can be substantively affected by the presence of production delay. Here we consider a mathematical model with bursty production of protein, a one-step production delay (the passage of which activates the protein), and feedback in the frequency of bursts. We specifically focus on examining the steady-state behaviour of the model in the slow-activation (i.e. large-delay) regime. Using a formal asymptotic approach, we derive an autonomous ordinary differential equation for the inactive protein that applies in the slow-activation regime. If the differential equation is monostable, the steady-state distribution of the inactive (active) protein is approximated by a single Gaussian (Poisson) mode located at the globally stable fixed point of the differential equation. If the differential equation is bistable (due to cooperative positive feedback), the steady-state distribution of the inactive (active) protein is approximated by a mixture of Gaussian (Poisson) modes located at the stable fixed points; the weights of the modes are determined from a WKB approximation to the stationary distribution. The asymptotic results are compared to numerical solutions of the chemical master equation.

## Introduction

Gene expression in individual cells involves the interaction of molecules which are present at low copy numbers (Eldar and Elowitz 2010; Munsky et al. 2012). The intrinsic noise generated by the low-copy-number reactions is passed down to the end product of gene expression, the protein, and results in temporal fluctuations and cell-to-cell heterogeneity of the protein copy number (Taniguchi et al. 2010; Suter et al. 2011). The production of proteins in bursts of multiple copies is one of the most important sources of protein variability (Singh et al. 2010; Dar et al. 2012). Specifically, bursty production accounts for super-Poissonian variability observed in protein copy numbers (Thattai and van Oudenaarden 2001).

Mathematical modelling has been proved useful in understanding the mechanisms of stochastic gene expression. The underlying probability distributions are typically defined as solutions to a specific master equation (Paulsson 2005; Veerman et al. 2018; Albert 2019). Explicit solutions to the master equation, especially at steady state, can be found for models with few components (Bokes et al. 2012; Zhou and Liu 2015) and/or with special structural properties (Kumar et al. 2015; Anderson and Cotter 2016). Generally, however, explicit solutions are unavailable or intractable and one resorts to stochastic simulation or seeks a numerical solution to a finite truncation of the master equation (Munsky and Khammash 2006; Borri et al. 2016; Gupta et al. 2017). An alternative approach, which often provides useful qualitative insights into the model behaviour, is based on reduction techniques such as quasi-steady-state (Srivastava et al. 2011; Kim et al. 2014; Plesa et al. 2019) and adiabatic reductions (Bruna et al. 2014; Popovic et al. 2016), piecewise-deterministic framework (Lin and Doering 2016; Lin and Buchler 2018), linear-noise approximation (Schnoerr et al. 2017; Modi et al. 2018), or moment closure (Singh and Hespanha 2007; Andreychenko et al. 2017; Gast et al. 2019).

Production delay is an inevitable part of gene expression (Monk 2003; Zavala and Marquez-Lago 2014; Bokes et al. 2018; Qiu et al. 2020). It can be caused by a number of mechanisms, e.g. transcriptional/translational elongation (Roussel and Zhu 2006), post-translational modification (Gedeon and Bokes 2012), or compartmental transport (Mor et al. 2010; Sturrock et al. 2017). The delay specifies the amount of time that needs to pass before a newly produced molecule can partake in its regulatory function (specifically in feedback). Delay can be fixed or randomly chosen from a distribution (Barrio et al. 2006; Lafuerza and Toral 2011; Gupta et al. 2014). Exponentially distributed delays are the simplest among distributed delays as they are realised by the passage of a single memoryless step. Erlang and phase-type distributions provide a wider family of distributed delays which can be generated by a finite network of memoryless states (Soltani et al. 2016). Previous results indicate that large one-step (exponential) and multi-step (Erlang/phase-type) delays reduce the super-Poissonian noise in a bursty protein down to Poissonian levels (Singh and Bokes 2012; Stoeger et al. 2016; Smith and Singh 2019). This effect is also seen experimentally with buffered noise in cytoplasmic mRNA levels compared to nuclear mRNA levels due to transport delays (Battich et al. 2015). Additional effects of the inclusion of a delay are observed if the protein regulates, via transcriptional feedback, its burst frequency. In case of negative feedback, delays of moderate size lead to an increase, rather than decrease in protein noise (Smith and Singh 2019). In case of non-cooperative positive feedback, noise-driven bimodal protein distributions, which are observed in the absence of delay, turn unimodal upon the inclusion of a distributed delay, and eventually converge to the Poissonian statistics as the delay increases (Borri et al. 2019). In case of cooperative positive feedback, the introduction of delay has been reported to enhance the stability of the modes of the protein distribution (Gupta et al. 2013; Feng et al. 2016; Kyrychko and Schwartz 2018).

In the paper we focus, for its relative simplicity, on the case of exponential delay. We refer to the delay as activation and distinguish between the inactive and active protein species. We will argue that in the limit of slow activation rates the model behaviour becomes deterministic at the level of the inactive protein. Behaviour of stochastic models near a deterministic limit can be interpreted using the large-deviation theory (Tsimring and Pikovsky 2001; Heymann and Vanden-Eijnden 2008; Kumar and Kulkarni 2019) and quantified by WKB asymptotic approximations (Schuss 2009; Bressloff 2014; Assaf and Meerson 2017). The WKB approach has been successfully applied to stochastic reaction kinetics systems with large molecule copy numbers (Hinch and Chapman 2005; Be’er and Assaf 2016; Yin and Wen 2019), fast switching of internal states (Newby 2012; Lin and Galla 2016), or a combination of both ( Newby and Chapman 2014, discussed at greater length in Sect. 7). Here we will use the WKB approximation to obtain reliable estimates of the stationary distribution of the active (and also the inactive) protein in the slow activation (large-delay) regime.

The paper is structured as follows. Section 2 introduces the stochastic model and its chemical master equation (CME). Section 3 seeks a WKB approximation to the stationary solution of the CME and formulates a specific algebraic problem that needs to be solved to determine the approximation. Section 4 solves the algebraic problem by analysing the phase plane of an associated dynamical system; a specific restriction of the dynamical system is thereby interpreted in terms of the emergent deterministic dynamics of the model in the slow-activation limit. Section 5 completes the calculation of the terms required for the WKB approximation. Section 6 develops the WKB approximation into tractable Gaussian/Poisson singleton/mixture approximations and compares them to the numerical solution of the CME. Section 7 concludes the paper with a discussion.

## Master equation

The paper is concerned with a reaction system involving the inactive and active protein species X and S which are subject to the production, activation, and decay reaction channels (Table 1). Each reaction is specified by its rate and reset map. The reaction rate, upon the multiplication by the length of an infinitesimally short time interval, gives the probability that the reaction will occur within the interval. The reset map is applied on the copy numbers of the two molecular species each time the reaction occurs.

**Table 1:** Reaction channels of the delayed feedback model with reaction species X (inactive protein) and S (active protein)

Name	Scheme	Rate	Reset map
Production	$\emptyset \rightarrow B \times \text {X}$	$\varepsilon ^{-1} f_s$	$X \rightarrow X + B$
Activation	$\text {X} \rightarrow \text {S}$	*X*	$X \rightarrow X - 1$,
			$$s \rightarrow \min \{s+1,s_\text {max}\}$$
Decay	$\text {S} \rightarrow \emptyset$	$\varepsilon ^{-1} s$	$s \rightarrow s - 1$

The copy number of the active protein is denoted by *s* and that of the inactive protein by *X* (we reserve the lower case for its concentration $x=\varepsilon X$, which is of the same order as *s*). The production burst rate $f_s$ is a function of *s*, which implements the feedback. The production burst size *B* is in general drawn from a prescribed random distribution. The parameter $\varepsilon \ll 1$ determines the discrepancy between the *O*(1) long timescale of slow activation and the $O(\varepsilon )$ short timescale of fast turnover of the active protein. The reset map of the activation channel ensures that *s* never exceeds an upper bound $s_\text {max}$

We are specifically interested in studying the model in the regime of slow activation. Making activation slow is equivalent to making all the remaining reactions fast: indeed, by Table 1, the activation rate is *O*(1), whereas the production and decay rates are $O(1/\varepsilon )$, where $\varepsilon \ll 1$ is a small dimensionless parameter. The aim of the paper is to find asymptotic approximations, valid for $\varepsilon \ll 1$, of the stationary behaviour of the model.

Let us talk through the specific forms of the reaction rates and reset maps of the individual reactions in Table 1. The production rate depends on the number *s* of active protein through a general (integer-valued) feedback response function $f_s$. The production reset map indicates that the number *X* of inactive proteins is increased by the size $B\ge 0$ of a production burst. Bursts sizes are drawn (independently of each other) from a distribution

1 $$\begin{aligned} P[B=j] = b_j,\quad j=0,1,2,\ldots \end{aligned}$$

The activation rate is proportional to the number of inactive proteins; the factor of proportionality is set to one without loss of generality. The activation reset map turns one inactive protein into an active protein if there is extra capacity for active protein ($s<{s_\text {max}}$); it removes an inactive protein without creating an active protein if there is no capacity ($s={s_\text {max}}$). The motivation for including the upper bound is technical: it allows for the use of finite-dimensional mathematical techniques. We will set ${s_\text {max}}$ to a value at which the decay rate exceeds, by a factor of two, the maximal rate of production; such a limit is reached rarely and its introduction affects the system dynamics negligibly. The decay rate is proportional to the number of active proteins; the decay reset map decreases the number of active proteins by one.

The probability $P = P(X, s, t)$ of having *X* molecules of inactive protein and *s* molecules of active protein (cf. Table 1) at time *t* satisfies the chemical master equation (CME)

2 $$\begin{aligned} \varepsilon \frac{\partial P}{\partial t} = f_s \left( \sum _{j=0}^\infty b_j {\mathbb {E}}^{-j}_{X} -1 \right) P + \varepsilon \left( {{\mathbb {E}}}_X {{\mathbb {E}}}^{-1}_s - 1\right) X P + \left( {\mathbb {E}}_s - 1\right) s P, \end{aligned}$$

in which ${\mathbb {E}}_X$ and ${\mathbb {E}}_s$ denote the van Kampen step operators (van Kampen 2006) in variables *X* and *s*, and ${\mathbb {E}}_X^{-1}$ and ${\mathbb {E}}_s^{-1}$ are their formal inverses.

The master equation (2) applies in the unbounded case ${s_\text {max}}=\infty$; in order to formulate one that is applicable to the ${s_\text {max}}<\infty$ case, it turns out to be helpful to use modified versions of the van Kampen step operators. For a sequence $v_s$ indexed by an integer $0\le s \le {s_\text {max}}$, we define the left- and right-shift operators by

3 $$\begin{aligned} {\mathcal {L}} v_s = \left\{ \begin{aligned}&v_{s+1}&\ \text {if } 0\le s< {s_\text {max}},\\&0&\ \text {if } s = {s_\text {max}}, \end{aligned} \right. \quad {\mathcal {R}} v_s = \left\{ \begin{aligned}&0&\ \text {if } s = 0,\\&v_{s-1}&\ \text {if } 0< s < {s_\text {max}},\\&v_{s-1} + v_s&\ \text {if } s= {s_\text {max}}. \end{aligned} \right. \end{aligned}$$

Away from the boundary $s={s_\text {max}}$, the left- and right-shift operators ${\mathcal {L}}$ and ${\mathcal {R}}$ are equal to the van Kampen step operator and its formal inverse, respectively. Let us discuss the meaning of the modifications of the operators that are made on the boundary $s={s_\text {max}}$. The left-shift operator is used below to describe the transfer of probability mass due to protein decay. The modification of the left-shift operator at the boundary $s={s_\text {max}}$ means there is no transfer of probability from the inadmissible state ${s_\text {max}}+1$. The right-shift operator is used below to describe the transfer of probability mass due to protein activation. The reset map of the activation reaction channel (Table 1) implies that states with ${s_\text {max}}-1$ as well as ${s_\text {max}}$ active protein molecules transfer into a state with ${s_\text {max}}$ molecules. Correspondingly, the right-shift operator returns at the boundary the sum of the ultimate and the penultimate terms of the original sequence.

In the bounded case ${s_\text {max}}< \infty$, the CME can be written as

4 $$\begin{aligned} \begin{aligned} \varepsilon \frac{\partial }{\partial t} P(X,s,t)&= f_s \sum _{j=0}^\infty b_j P(X-j,s,t) + \varepsilon (X+1) {\mathcal {R}} P(X+1, s, t)\\&\quad + {\mathcal {L}} s P(X,s, t) - (f_s + \varepsilon X + s) P(X,s,t), \end{aligned} \end{aligned}$$

in which we use the operator formalism (3) in the variable *s*, but the shifts in the variable *X* are made explicit. We thereby tacitly understand, as is customary in analyses of CMEs, that the probability of having a negative number of species *X* is equal to zero.

In the slow-activation regime ($\varepsilon \ll 1$), the inactive protein is present at $O(\varepsilon ^{-1})$ large copy numbers. In order to measure the abundance of the species on an *O*(1) scale, we define

5 $$\begin{aligned} X = \frac{x}{\varepsilon },\quad P(X,s,t) = p(x,s,t). \end{aligned}$$

We refer to the new variable *x*, which becomes in the limit of $\varepsilon \rightarrow 0$ a continuous quantity, as the concentration of the inactive protein. Inserting (5) into (4), we obtain

6 $$\begin{aligned} \begin{aligned} \varepsilon \frac{\partial }{\partial t} p(x,s,t)&= f_s \sum _{j=0}^\infty b_j p(x-\varepsilon j,s,t) + (x+\varepsilon ) {\mathcal {R}} p(x+\varepsilon , s, t)\\&\quad + {\mathcal {L}} s p(x,s, t) - (f_s + x + s) p(x,s,t). \end{aligned} \end{aligned}$$

Equating the derivative in (6) to zero, we arrive at a bivariate difference equation

7 $$\begin{aligned} 0 = f_s \sum _{j=0}^\infty b_j p(x-\varepsilon j,s) + (x+\varepsilon ) {\mathcal {R}} p(x+\varepsilon , s) + {\mathcal {L}} s p(x,s) - (f_s + x + s) p(x,s) \end{aligned}$$

for the steady-state distribution *p*(*x*, *s*). Below we seek an asymptotic approximation as $\varepsilon \rightarrow 0$ to the (normalised) solution of (7).

## Expansion

We seek a solution to (7) in the WKB form

8 $$\begin{aligned} p(x,s;\varepsilon ) = \left( r_s^0(x) + \varepsilon r_s^1(x) + O(\varepsilon ^2)\right) \text {e}^{-\frac{\varPhi (x)}{\varepsilon }}, \end{aligned}$$

where $r_s^0(x)>0$ and $r_s^1(x)$ give the first two terms in the asymptotic expansion in the powers of $\varepsilon$. The variable *s* is placed in the subscript to emphasise its discreteness (contrasting it with the continuous nature of *x* in the limit of $\varepsilon \rightarrow 0$). The function $\varPhi (x)$ in the exponent of (8) is referred to as the WKB potential.

Below we develop, by means of (8), the individual terms of the difference Eq. (7) into asymptotic expansions of up to the second order. This is a mechanistic but laborious exercise. Therefore we suggest that, on first reading, the reader focus their attention on the leading-order terms in the expansions; these are sufficient for calculating the potential $\varPhi (x)$, which plays the central role in the analysis.

For the first term in Eq. (7) we find

$$\begin{aligned}&\sum _{j=0}^\infty b_j p(x-\varepsilon j, s; \varepsilon ) \\&\quad = \sum _{j=0}^\infty b_j \left( r_s^0(x-\varepsilon j) + \varepsilon r_s^1(x-\varepsilon j) +O(\varepsilon ^2) \right) \text {e}^{-\varPhi (x-j\varepsilon )/\varepsilon }\\&\quad = \text {e}^{-\frac{\varPhi (x)}{\varepsilon }} \sum _{j=0}^\infty b_j \text {e}^{j\theta } \left( r_s^0(x) - \varepsilon j r_s^{0\,\prime }(x) + \varepsilon r_s^1(x) + O(\varepsilon ^2) \right) \left( 1 - \frac{\varepsilon j^2 \varPhi ''(x)}{2} + O(\varepsilon ^2) \right) \\&\quad \sim \text {e}^{-\frac{\varPhi (x)}{\varepsilon }} \Big ( r_s^0(x) M(\theta ) + \varepsilon \Big [ r_s^1(x) M(\theta ) - r_s^{0\,\prime }(x) M'(\theta ) -\frac{1}{2}\varPhi ''(x) r_s^0(x) M''(\theta ) \Big ]\Big ), \end{aligned}$$

where

9 $$\begin{aligned} \theta = \varPhi '(x),\quad M(\theta ) = \sum _{j=0}^\infty b_j \text {e}^{j\theta } \end{aligned}$$

are the potential derivative and the moment generating function of the burst-size probability distribution (1), respectively.

The second term in Eq. (7) is developed into

$$\begin{aligned}&(x+\varepsilon ) {\mathcal {R}} p(x+\varepsilon ,s;\varepsilon ) \\&\quad = (x+\varepsilon ) \left( {\mathcal {R}} r_s^0(x+\varepsilon ) + \varepsilon {\mathcal {R}} r_s^1(x+\varepsilon ) + O(\varepsilon ^2) \right) \text {e}^{-\varPhi (x+\varepsilon )/\varepsilon }\\&\quad = \text {e}^{-\frac{\varPhi (x)}{\varepsilon }-\theta } (x + \varepsilon ) \left( {\mathcal {R}} r_s^0(x) + \varepsilon [ {\mathcal {R}} r_s^{0\,\prime }(x) + {\mathcal {R}} r_s^1(x)] + O(\varepsilon ^2) \right) \left( 1 - \frac{\varepsilon \varPhi ''(x)}{2} + O(\varepsilon ^2) \right) \\&\quad \sim \text {e}^{-\frac{\varPhi (x)}{\varepsilon } - \theta } \left( x {\mathcal {R}} r_s^0(x) + \varepsilon \left[ \left( 1 - \frac{\varPhi ''(x) x}{2} \right) {\mathcal {R}} r_s^0(x) + x\left( {\mathcal {R}} r_s^{0\,\prime }(x) + {\mathcal {R}} r_s^1(x)\right) \right] \right) . \end{aligned}$$

The remaining terms in (7) are easy to expand.

We insert the WKB ansatz (8) into (7), expand the individual terms of the equation as done above, and collect terms of same order; at the leading order this yields

10 $$\begin{aligned} {\mathcal {A}} r_s^0(x) = 0, \end{aligned}$$

where

11 $$\begin{aligned} {\mathcal {A}} v_s = \text {e}^{-\theta } x {\mathcal {R}} v_s + {\mathcal {L}} s v_s - (f_s (1- M(\theta )) + x + s) v_s \end{aligned}$$

is a linear operator acting on sequences $v_s$ defined for $0\le s \le {s_\text {max}}$. Such sequences can be represented by $({s_\text {max}}+1)$-dimensional column vectors $\varvec{v}=(v_0,v_1, \ldots , v_{{s_\text {max}}})^\intercal$, and the linear operator ${\mathcal {A}}$ as a square matrix $\varvec{A}$ of order ${s_\text {max}}+1$. Here and below, we will go back and forth between the operator–sequence and the matrix–vector notations, using that which expresses a given formula more succinctly.

The matrix $\varvec{A}$ is tridiagonal. On the main diagonal it has the sequence $- f_s (1- M(\theta )) - x - s$, where $0\le s < {s_\text {max}}$, except for the last diagonal element, which is given by $- f_{{s_\text {max}}} (1-M(\theta )) - x(1-\text {e}^{-\theta }) - {s_\text {max}}$. On the upper diagonal it has the sequence $1,2,\ldots , {s_\text {max}}$; the elements of the lower diagonal are all equal to $\text {e}^{-\theta }x$. It looks like12The matrix $\varvec{A}=\varvec{A}(x,\theta )$ — just like the associated operator ${\mathcal {A}}={\mathcal {A}}(x,\theta )$ — depends on the protein concentration *x* and the (yet unknown) potential derivative $\theta =\varPhi '(x)$.

Equation (10) can be written in a matrix form as

13 $$\begin{aligned} \varvec{A}(x,\theta )\varvec{r}^0(x) = \varvec{0}, \end{aligned}$$

where $\varvec{r}^0(x) = (r_0^0(x),r_1^0(x),\ldots ,r_{{s_\text {max}}}^0(x))^\intercal$ is required to be a positive vector (i.e. a vector with only positive elements). Denote by

14 $$\begin{aligned} H(x,\theta ) = \lambda _1(\varvec{A}(x,\theta )) \end{aligned}$$

the eigenvalue with largest real part (Bressloff and Newby 2014), which we refer to hereafter as the principal eigenvalue. The matrix $\varvec{A}$ does not have any negative off-diagonal elements; the Perron–Frobenius theorem implies that the principal eigenvalue is real, and its right and left eigenvectors (referred to as principal eigenvectors) are real and positive. The right eigenvectors of non-principal eigenvalues, being orthogonal to the left principal eigenvector, cannot be positive. Therefore, Eq. (13) is solvable in $\varvec{r}^0(x)>0$ if and only if

15 $$\begin{aligned} H(x,\theta ) = 0 \end{aligned}$$

holds. In what follows, we show that the zero-level set (15) includes a graph of a function $\theta =\theta (x)$, which forms the derivative (9) of the desired potential. In order to characterise the level sets of the function $H(x,\theta )$, it is useful to consider the associated Hamiltonian system of differential equations.

## Hamiltonian system

Differentiating with respect to *x* Eq. (15) in which $\theta =\theta (x)$ yields

$$\begin{aligned} \frac{\partial H}{\partial x} + \frac{\partial H}{\partial \theta }\frac{{\mathrm{d}}\theta }{{\mathrm{d}}x}=0, \end{aligned}$$

i.e.

16 $$\begin{aligned} \frac{{\mathrm{d}}\theta }{{\mathrm{d}}x} = - \frac{\frac{\partial H}{\partial x} (x,\theta )}{\frac{\partial H}{\partial \theta } (x,\theta )}. \end{aligned}$$

The non-autonomous differential Eq. (16) is equivalent to the system of two autonomous differential equations

17 $$\begin{aligned} {\dot{x}} = \frac{\partial H}{\partial \theta } (x,\theta ),\quad {\dot{\theta }} = -\frac{\partial H}{\partial x} (x,\theta ), \end{aligned}$$

in which the dot represents the time derivative. The system is Hamiltonian: its trajectories form the level sets of (14). We are specifically interested in the zero set (15). Borrowing terminology from the Hamiltonian formalism, we refer to the variable $\theta$ as the conjugate momentum.

In order to solve system (17), we need to evaluate the right-hand sides. For this purpose it is useful to express the Hamiltonian (14) as

18 $$\begin{aligned} H = \varvec{u}^\intercal \varvec{A} \varvec{v}, \end{aligned}$$

where $\varvec{u}>0$ and $\varvec{v}>0$ are the left and right eigenvectors corresponding to the principal eigenvalue *H*, i.e.

19 $$\begin{aligned} \varvec{u}^\intercal \varvec{A} = H \varvec{u}^\intercal ,\quad \varvec{A} \varvec{v} = H \varvec{v}, \end{aligned}$$

which additionally satisfy

20 $$\begin{aligned} \varvec{u}^\intercal \varvec{v} = 1, \quad \sum _{s=0}^{s_\text {max}}v_s = 1. \end{aligned}$$

Conditions (20) can be met by a suitable choice of multiplicative constants.

The principal eigenvectors $\varvec{u}$ and $\varvec{v}$, just like $\varvec{A}$ and the principal eigenvalue *H*, depend on the protein concentration *x* and the conjugate momentum $\theta$. Differentiating (18) with respect to *x* yields

21 $$\begin{aligned} \frac{\partial H}{\partial x}&= \varvec{u}^\intercal \frac{\partial \varvec{A}}{\partial x} \varvec{v} + \frac{\partial \varvec{u}^\intercal }{\partial x} \varvec{A} \varvec{v} + \varvec{u}^\intercal \varvec{A} \frac{\partial \varvec{v}}{\partial x} \nonumber \\&= \varvec{u}^\intercal \frac{\partial \varvec{A}}{\partial x} \varvec{v} + H \frac{\partial \varvec{u}^\intercal \varvec{v}}{\partial x} \nonumber \\&= \varvec{u}^\intercal \frac{\partial \varvec{A}}{\partial x} \varvec{v}, \end{aligned}$$

where the first equality applies the product rule, the second follows by (19), while the third is due to (20). Differentiating (18) with respect to $\theta$ and simplifying as before gives

22 $$\begin{aligned} \frac{\partial H}{\partial \theta } = \varvec{u}^\intercal \frac{\partial \varvec{A}}{\partial \theta } \varvec{v}. \end{aligned}$$

The derivatives of $\varvec{A}$ in (21)–(22) are matrix representations of the derivatives

23 $$\begin{aligned} \frac{\partial {\mathcal {A}}}{\partial x} v_s = \text {e}^{-\theta } {\mathcal {R}} v_s - v_s,\quad \frac{\partial {\mathcal {A}}}{\partial \theta } v_s = M'(\theta ) f_s v_s -\text {e}^{-\theta } x {\mathcal {R}} v_s \end{aligned}$$

of the operator ${\mathcal {A}}$ (11). Before analysing the entire phase plane of system (17), we first characterise its flow on the *x*-axis and interpret it in terms of the model dynamics.

### Deterministic rate equation

The matrix $\varvec{A}(x,0)$ simplifies, owing to the relation $M(0)=1$, to a Markovian transition matrix of a birth–death process with state space $\{0,1,\ldots ,{s_\text {max}}\}$, a constant birth rate *x* (as long as $s<{s_\text {max}}$), and a linear death rate *s*. In our context, births correspond to activation (from a constant source of inactive protein), and deaths correspond to decay of the active protein. In queueing theory, which identifies births and deaths with customer arrivals and departures, such a process is referred to as the $M/M/{s_\text {max}}/{s_\text {max}}$ server with memoryless arrival and service times, ${s_\text {max}}$ servers, and no queue (Gross 2008).

The transition matrix $\varvec{A}(x,0)$ of the $M/M/{s_\text {max}}/{s_\text {max}}$ server has the principal eigenvalue and eigenvectors given by

24 $$\begin{aligned} H(x,0)=0,\quad \varvec{u}(x,0) = \varvec{1},\quad \varvec{v}(x,0) = \varvec{\rho }(x), \end{aligned}$$

in which $\varvec{1}=(1,1,\ldots ,1)^\intercal$ is an $({s_\text {max}}+1)$-dimensional vector of ones and $\varvec{\rho }(x)=(\rho _0(x),\rho _1(x),\ldots ,\rho _{s_\text {max}}(x))^\intercal$ is the process’s stationary distribution; this is given by the truncated Poisson distribution with location parameter *x* (Gross 2008),

25 $$\begin{aligned} \rho _s(x) = {\mathcal {N}}(x) \frac{x^s}{s!},\quad s = 0,1,\ldots ,{s_\text {max}}, \end{aligned}$$

where

26 $$\begin{aligned} {\mathcal {N}}(x) = \left( \sum _{s=0}^{{s_\text {max}}} \frac{x^s}{s!} \right) ^{-1} \end{aligned}$$

is the normalisation constant. We remark that the probability of the server running at full capacity, which is returned by (25) at $s={s_\text {max}}$, goes under the name of Erlang’s loss formula (Gross 2008).

Inserting (24) and (23) into (21) yields

27 $$\begin{aligned} \frac{\partial H}{\partial x} (x,0)&= \varvec{1}^\intercal \frac{\partial \varvec{A}}{\partial x} (x,0) \varvec{\rho }(x) = \sum _{s=0}^{s_\text {max}}\frac{\partial {\mathcal {A}}}{\partial x} (x,0) \rho _s(x)\nonumber \\&= \sum _{s=0}^{s_\text {max}}{\mathcal {R}} \rho _s(x) - \sum _{s=0}^{s_\text {max}}\rho _s(x) = 0, \end{aligned}$$

in which we used that the product of a row vector of ones and a column vector is equal to the sum of the column vector’s elements and that the right-shift operator ${\mathcal {R}}$ (3) preserves the sum of vector elements. Inserting (24) and (23) into (22) we find, after similar simplifications, that

28 $$\begin{aligned} \frac{\partial H}{\partial \theta } (x,0)&= \varvec{1}^\intercal \frac{\partial \varvec{A}}{\partial \theta } (x,0) \varvec{\rho }(x) = \sum _{s=0}^{s_\text {max}}\frac{\partial {\mathcal {A}}}{\partial \theta } (x,0) \rho _s(x)\nonumber \\&= M'(0)\sum _{s=0}^{s_\text {max}}f_s \rho _s(x) - x \sum _{s=0}^{s_\text {max}}{\mathcal {R}} \rho _s(x)\nonumber \\&= \langle B \rangle {\bar{f}}(x) - x, \end{aligned}$$

where

29 $$\begin{aligned} \langle B \rangle = M'(0) = \sum _{j=0}^\infty j b_j \end{aligned}$$

is the mean burst size and

30 $$\begin{aligned} {\bar{f}}(x) = \sum _{s=0}^{s_\text {max}}f_s \rho _s(x) \end{aligned}$$

is the expectation of $f_s$ with respect to the truncated Poisson distribution (25).

Inserting (27) into (17), we find that ${\dot{\theta }} = 0$ if $\theta =0$: the *x*-axis is an invariant set of the Hamiltonian system (17). Inserting (28) into (17), we find that on the invariant set $\theta =0$ the Hamiltonian system reduces to the rate equation

31 $$\begin{aligned} {\dot{x}} = \langle B \rangle {\bar{f}}(x) - x. \end{aligned}$$

The Hamiltonian system (17) thus comprises, and extends by an additional dimension in $\theta$, the concentration dynamics given by (31). The rate equation (31) represents a deterministic reduction of the delayed feedback model. This can be understood intuitively by invoking a quasi-steady-state (QSS) approximation (Rao and Arkin 2003). On $O(\varepsilon )$-short timescales, the inactive protein varies little and slowly, and its concentration *x* is nearly constant; on the other hand, the active protein is noisy and fast, and its copy number *s* evolves like an $M/M/{s_\text {max}}/{s_\text {max}}$ server, equilibrating to the QSS distribution (25). On *O*(1)-long timescales, the inactive protein is produced with an effective burst rate (30) which is obtained by averaging the instantaneous burst rate with respect to the QSS distribution. Multiplying the effective burst rate by the expected burst size and subtracting the linear activation rate yields the emergent deterministic dynamics (31).

**Fig. 1:**
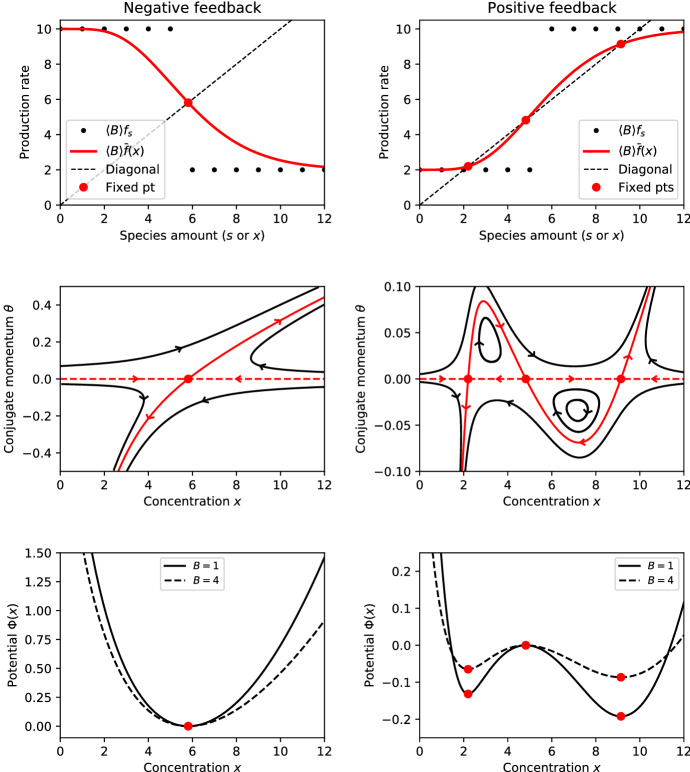
Top: The instantaneous production rate $\langle B \rangle f_s$ (black dots) and the QSS-averaged production rate $\langle B \rangle {\bar{f}}(x)$ (red curve). Centre: Phase plane of the Hamiltonian system (17). The heteroclinic orbits (shown in red) form the zero set (15). The nontrivial portion of the zero set (solid red curve) defines the derivative of the WKB potential $\theta =\varPhi '(x)$. The trivial portion of the zero set ($\theta =0$; dashed red line) is the phase line of the rate equation (31). Bottom: The WKB potential $\varPhi (x)$. Red circles in all panels: The fixed points of the QSS-averaged production rate, the saddles of the Hamiltonian system, and the extrema of the potential coincide. Parametric values: The upper bound on *S* is ${s_\text {max}}=20$. The feedback threshold is $s_\text {thresh}=6$. The burst size is fixed to $B=1$ except for the dashed curve, bottom panels, where it is fixed to $B=4$. Negative feedback examples use $a_0=10$, $a_1=2$, except for the dashed curve, bottom left panel, which uses $a_0=2.5$, $a_1=0.5$. Positive feedback examples use $a_0=2$, $a_1=10$, except for the dashed curve, bottom right panel, which uses $a_0=0.5$, $a_1=2.5$ (color figure online)

Although the main contribution to ${\bar{f}}(x)$ comes from the values of $f_s$ whose argument *s* is close to *x*, the value of ${\bar{f}}(x)$ is determined by all values of $f_s$. Due to the contributions of neighbouring terms, any sharp features of $f_s$ are smoothed out, or “mollified”, in the function ${\bar{f}}(x)$; for example, a step function (Gedeon et al. 2017; Crawford-Kahrl et al. 2019)

32 $$\begin{aligned} f_s = \left\{ \begin{aligned}&a_0&\ \text {if } s < s_\text {thresh},\\&a_1&\ \text {if } s \ge s_\text {thresh} \end{aligned} \right. \end{aligned}$$

turns into a smooth sigmoid function by the application of (30); see Fig. 1, top panels.

By (24), the *x*-axis belongs to the zero-level set (15). However, since $\theta =0$ cannot serve as the derivative of an appropriate potential, we need to look for a different branch $\theta =\varPhi '(x)$ of solutions to (15). The nontrivial branch is found by linearising the Hamiltonian system around its steady states on the *x*-axis.

### Phase-plane analysis

A point $(x_*,0)$, where $x_*$ is any of the fixed points of (31), is also a steady state of the full Hamiltonian system (17). The linearisation matrix is given by

33 $$\begin{aligned} \varvec{J} = \left. \begin{pmatrix} \frac{\partial ^{2} H}{\partial x \partial \theta } &{} \frac{\partial ^{2} H}{\partial \theta ^{2}}\\ 0 &{} -\frac{\partial ^{2} H}{\partial x \partial \theta } \end{pmatrix}\right| _{x=x_*, \theta =0,} \end{aligned}$$

in which the second derivative of *H* with respect to *x* is immediately seen to be zero because of (27).

It follows that $(x_*,0)$ is a saddle of (17) with eigenvalues

34 $$\begin{aligned} \lambda _{1,2}(\varvec{J}) = \pm \frac{\partial ^2 H}{\partial x\partial \theta } \end{aligned}$$

and eigenvectors

35 $$\begin{aligned} \text {Ker}(\varvec{J}-\lambda _1(\varvec{J})) \ni (1,0)^\intercal ,\quad \text {Ker}(\varvec{J}-\lambda _2(\varvec{J})) \ni \left( 1, -2 \left( \frac{\partial ^{2}H}{\partial \theta ^{2}}\right) ^{-1} \frac{\partial ^{2} H}{\partial x\partial \theta } \right) ^\intercal , \end{aligned}$$

in which the derivatives of the Hamiltonian are evaluated at $(x_*,0)$. One can show that

36 $$\begin{aligned} \frac{\partial ^2 H}{\partial x\partial \theta } (x_*,0)&= \langle B \rangle {\bar{f}}'(x_*) - 1, \end{aligned}$$

37 $$\begin{aligned} \frac{\partial ^{2} H}{\partial \theta ^{2}} (x_*,0)&= x_*\left( 1 + \frac{\langle B^2 \rangle }{\langle B \rangle }\right) + 2 \varvec{1}^\intercal \frac{\partial \varvec{A}}{\partial \theta } (x_*,0) \tilde{\varvec{v}}, \end{aligned}$$

where $\tilde{\varvec{v}}$ is a solution to $\varvec{A}(x_*,0) \tilde{\varvec{v}} = - \frac{\partial \varvec{A}}{\partial \theta } (x_*,0) \varvec{\rho }(x_*)$. Equation (36) is an immediate consequence of (28). Equation (37) is derived in the “Appendix”. The derivative of the effective production rate (30) satisfies

38 $$\begin{aligned} {\bar{f}}'(x) = \sum _{s=0}^{{s_\text {max}}- 1} (f_{s+1} - f_s) \rho _s(x) + \left( {\bar{f}}(x) - f_{{s_\text {max}}}\right) \rho _{s_\text {max}}(x). \end{aligned}$$

Equations (36)–(38) provide a practical numerical recipe for calculating the nontrivial eigenvector (35) of the Hamiltonian system linearisation.

The trajectories emanating from a saddle $(x_*,0)$ along the direction of the eigenvector $(1,0)^\intercal$ form the trivial branch $\theta =0$ of the zero set (15) (Fig. 1, central panels, dashed red line). The trajectories emanating from the saddle along the nontrivial eigenvector (35) form the nontrivial branch of the zero set (15) (Fig. 1, central panels, solid red curve). The nontrivial branch constitutes the sought-after WKB potential derivative $\theta =\varPhi '(x)$. Given that the potential derivative has the opposite sign to the deterministic flow on the *x*-axis, we have

39 $$\begin{aligned} \frac{\text {d}}{\text {d}t} \varPhi (x(t)) = \varPhi '(x(t)) {\dot{x}}(t) < 0 \end{aligned}$$

for non-stationary solutions to (31). Therefore, the potential $\varPhi (x)$ is a Lyapunov function of the rate equation (31), possessing local minima (maxima) where (31) has stable (unstable) fixed points (Fig. 1, bottom panels).

The potential carries additional information about the noise in the model that the rate equation does not: specifically, the rate equation depends only on the product of burst size and burst frequency, remaining the same if the burst size is multiplied by the same factor as the burst frequency is divided by; the potential, on the other hand, becomes flatter as the system becomes more bursty (Fig. 1, bottom panels, dashed curves). This observation is consistent with an intuition that bursty production enhances noise and the chance to escape potential wells.

**Fig. 2:**
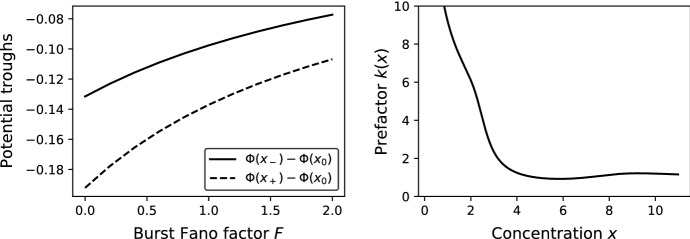
Left: The depths of potential troughs as functions of the Fano factor of binomially-distributed burst sizes with mean $\langle B \rangle =1$. Right: The WKB prefactor in the non-bursty case ($B=1$). Parameter values for both panels: ${s_\text {max}}=20$; $s_\text {thresh}=6$; $a_0=2$; $a_1=10$

In order to investigate the impact of distributed burst sizes, we consider burst distributions with moment generating functions (MGFs)

40 $$\begin{aligned} M(\theta ) = \left( F + (1-F) \text {e}^\theta \right) ^\frac{\langle B \rangle }{1 - F} \end{aligned}$$

parametrised by the mean $\langle B \rangle >0$ and the Fano factor $F \ge 0$ (the variance-to-mean ratio). For $F=0$, definition (40) simplifies to $M(\theta )=\text {e}^{\langle B \rangle \theta }$, which is the MGF of a fixed burst size $B=\langle B\rangle$ (assuming that $\langle B \rangle$ is an integer). For $0<F<1$, formula (40) gives the MGF of a binomial distribution. As $F\rightarrow 1$, the right-hand side of (40) tends to $\text {e}^{\langle B \rangle (\text {e}^\theta - 1)}$, which gives the MGF of the Poisson distribution. For $F>1$, expression (40) is the MGF of the negative binomial distribution; specifically, for $F=1+\langle B \rangle$, it reduces to the MGF $M(\theta )=(1+\langle B \rangle (1-\text {e}^\theta ))^{-1}$ of the widely used geometric distribution of burst sizes (McAdams and Arkin 1997). Fixing the burst size mean $\langle B \rangle$ and varying the Fano factor *F*, the troughs of a double-well potential become shallower (Fig. 2, left).

## Prefactor

In order to complete the WKB approximation (8) at the leading order, we express the solution to the linear Eq. (10) in terms of the $l^1$-normalised nullvector (19) as

41 $$\begin{aligned} r^0_s(x) = k(x) w_s(x),\quad w_s(x) = v_s(x,\varPhi '(x)), \end{aligned}$$

where *k*(*x*) is an *s*-independent prefactor.

The calculation of the prefactor requires to consult the second-order terms in the WKB expansion of the master equation. Recall that in Sect. 3 we inserted the WKB ansatz (8) into the master equation (7), expanded (the individual terms of the equation) up to the second order, and collected the first-order terms. Collecting the second-order terms yields

$$\begin{aligned} {\mathcal {A}} r_s^1(x)&- f_s \left( M'(\theta ) r_s^{0\,\prime }(x) + \frac{1}{2} \varPhi ''(x) M''(\theta ) r_s^0(x)\right) \\&\quad + \text {e}^{-\theta }\left[ \left( 1 - \frac{1}{2} \varPhi ''(x) x \right) {\mathcal {R}} r_s^0(x) + x {\mathcal {R}} r_s^{0\,\prime }(x) \right] = 0. \end{aligned}$$

Inserting the factorisation (41) into the above equation yields

42 $$\begin{aligned} {\mathcal {A}} r_s^1(x) = k'(x) \alpha _s(x) + k(x) \beta _s(x), \end{aligned}$$

where

$$\begin{aligned} \alpha _s(x)&= M'(\theta ) f_s w_s(x) - \text {e}^{-\theta } x {\mathcal {R}} w_s(x), \\ \beta _s(x)&= M'(\theta ) f_s w_s'(x) + \frac{1}{2} \varPhi ''(x) M''(\theta ) f_s w_s(x)\\&\quad - \text {e}^{-\theta } \left[ \left( 1 - \frac{1}{2} \varPhi ''(x) x \right) {\mathcal {R}} w_s(x) + x {\mathcal {R}} w_s'(x) \right] . \end{aligned}$$

In order that Eq. (42) be solvable in $r_s^1(x)$, its right-hand side must be orthogonal to the left nullvector $l_s(x)=u_s(x,\varPhi '(x))$ of ${\mathcal {A}}={\mathcal {A}}(x,\varPhi '(x))$, i.e.

$$\begin{aligned} k'(x) \sum _{s=0}^{s_\text {max}}l_s(x) \alpha _s(x) + k(x) \sum _{s=0}^{s_\text {max}}l_s(x) \beta _s(x) = 0. \end{aligned}$$

Integrating the above linear homogeneous first-order equation in *k*(*x*) yields

$$\begin{aligned} k(x) = \exp \left( - \int \frac{ \sum _{s=0}^{s_\text {max}}l_s(x) \beta _s(x) }{ \sum _{s=0}^{s_\text {max}}l_s(x) \alpha _s(x) } \text {d}x \right) . \end{aligned}$$

The dependence of the prefactor *k*(*x*) on the protein concentration *x* is exemplified in Fig. 2, right.

## Mixture approximations

Combining (8) and (41), we express the WKB approximation to the joint distribution $p(x,s;\varepsilon )$ of the inactive protein concentration *x* and the active protein copy number *s* in the form of

43 $$\begin{aligned} p(x,s;\varepsilon ) \sim k(x) w_s(x) \text {e}^{-\frac{\varPhi (x)}{\varepsilon }}, \quad \varepsilon \ll 1, \end{aligned}$$

where *k*(*x*), $w_s(x)$ and $\varPhi (x)$ are independent of $\varepsilon$ and satisfy $k(x)>0$, $w_s(x)>0$, and $\sum _{s=0}^{s_\text {max}}w_s(x) = 1$.

Expressing the marginal distribution of *x* and the conditional distribution of *s* as

$$\begin{aligned} p(x,\cdot \,;\varepsilon ) = \sum _{s=0}^{s_\text {max}}p(x,s;\varepsilon ) \sim k(x) \text {e}^{-\frac{\varPhi (x)}{\varepsilon }},\quad p(s | x;\varepsilon ) = \frac{p(x,s;\varepsilon )}{p(x,\cdot \,;\varepsilon )} \sim w_s(x), \end{aligned}$$

we refer to $w_s(x)$ as the WKB conditional distribution of *s*, and note that the potential $\varPhi (x)$ and the prefactor *k*(*x*) constitute the WKB marginal distribution of *x*.

Dominant contributions to the protein distribution (43) come from the neighbourhoods of the minima of the potential $\varPhi (x)$, where the potential can be approximated by parabolas. In the monostable regime of the rate equation (31), when the potential has a global minimum at $x_0$, doing so leads to

44 $$\begin{aligned} p(x,s;\varepsilon ) \sim k(x_0) w_s(x_0) \text {exp}\left( -\frac{\varPhi (x_0)}{\varepsilon } -\frac{\varPhi ''(x_0)(x - x_0)^2}{2\varepsilon } \right) , \end{aligned}$$

whereas in the bistable regime with two minima $x_-$ and $x_+$ we obtain

45 $$\begin{aligned} \begin{aligned} p(x,s;\varepsilon )&\sim k(x_-) w_s(x_-) \text {exp}\left( -\frac{\varPhi (x_-)}{\varepsilon } -\frac{\varPhi ''(x_-)(x - x_-)^2}{2\varepsilon } \right) \\&\quad + k(x_+) w_s(x_+) \text {exp}\left( -\frac{\varPhi (x_+)}{\varepsilon } -\frac{\varPhi ''(x_+)(x - x_+)^2}{2\varepsilon } \right) . \end{aligned} \end{aligned}$$

Combining (41) and (24), we find that at critical points of the WKB potential the WKB conditional distribution of *s* satisfies

46 $$\begin{aligned} w_s(x_*) = v_s(x_*,0) = \rho _s(x_*), \quad 0\le s \le {s_\text {max}}, \quad x_*\in \{x_-,x_0,x_+\}, \end{aligned}$$

where $\rho _s(x)$ is the QSS Poisson distribution defined by (25)–(26). Interestingly, conditioning on non-critical points of the potential leads to WKB conditional distributions of *s* that differ from the QSS ones. Arguably, this disagreement arises because the simplifying QSS assumption of a fixed inactive protein concentration is invalidated by making a large and improbable deviation from a fixed point. Nevertheless, the contribution of non-QSS conditional distributions towards the total distribution of *s* is seen to be negligible by the application of the parabolic approximations (44)–(45).

**Fig. 3:**
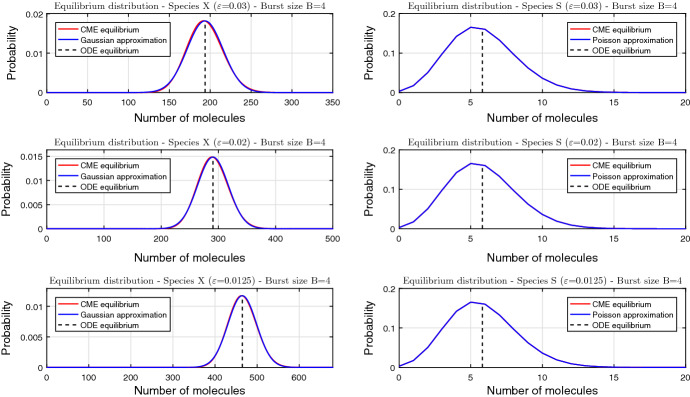
Steady-state distributions for negative feedback and fixed burst size $B=4$ obtained by numerical solution (red) and asymptotic approximation (blue). Panel columns refer to the two protein species; panel rows refer to distinct values of $\varepsilon$. The dashed lines indicate the locations of the stable fixed points (in units of molecules in the left column, and units of concentration in the right column) of the deterministic equation (31). The step function (32) parameters are $s_\text {thresh}=6$, $a_0=2.5$, $a_1=0.5$ (color figure online)

Inserting (46) into (44) and replacing (*x*, *s*)-independent factors by an appropriate normalisation constant yields

47 $$\begin{aligned} p(x,s;\varepsilon ) \sim \rho _s(x_0) \times \sqrt{\frac{\varPhi ''(x_0)}{2\pi \varepsilon }} \exp \left( -\frac{\varPhi ''(x_0)(x-x_0)^2}{2\varepsilon }\right) . \end{aligned}$$

Integrating (47) over *x* implies that *s* follows a truncated Poisson distribution with location parameter $x_0$ (Fig. 3, right panels); summing (47) over *s* implies that *x* follows a Gaussian distribution with mean $x_0$ and variance $\varepsilon /\varPhi ''(x_0)$. Linearly transforming concentration into copy number by means of $X=x/\varepsilon$, we find that the latter also follows a Gaussian distribution with mean $x_0/\varepsilon$ and variance $1/\varepsilon \varPhi ''(x_0)$ (Fig. 3, left panels).

**Fig. 4:**
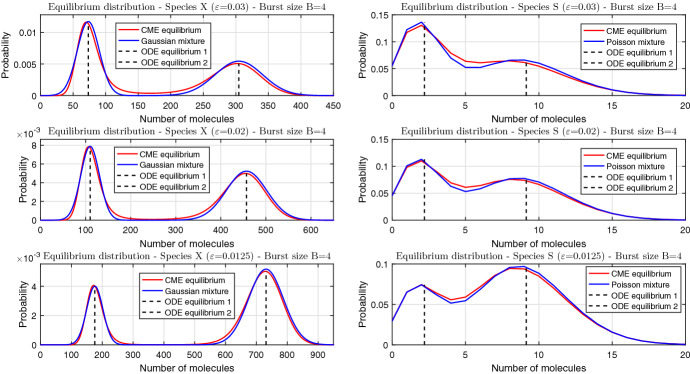
Steady-state distributions for the case of positive feedback and fixed burst size $B=4$ obtained by numerical solution (red) and asymptotic approximation (blue). Panel columns refer to the two protein species; panel rows refer to distinct values of $\varepsilon$. The dashed lines indicate the locations of the stable fixed points (in units of molecules in the left column, and units of concentration in the right column) of the deterministic equation (31). The step function (32) parameters are $s_\text {thresh}=6$, $a_0=0.5$, $a_1=2.5$ (color figure online)

Inserting (46) into (45) leads to

48 $$\begin{aligned} \begin{aligned} p(x,s;\varepsilon )&\sim \omega _- \times \rho _s(x_-) \times \sqrt{\frac{\varPhi ''(x_-)}{2\pi \varepsilon }}\exp \left( -\frac{\varPhi ''(x_-)(x-x_-)^2}{2\varepsilon }\right) +\\&\quad \quad \omega _+ \times \rho _s(x_+) \times \sqrt{\frac{\varPhi ''(x_+)}{2\pi \varepsilon }}\exp \left( -\frac{\varPhi ''(x_+)(x-x_+)^2}{2\varepsilon }\right) \end{aligned} \end{aligned}$$

with weights given by

49 $$\begin{aligned} \omega _\pm = \frac{C k(x_\pm )}{\sqrt{\varPhi ''(x_\pm )}} \exp \left( -\frac{\varPhi (x_\pm )}{\varepsilon } \right) , \end{aligned}$$

in which the constant *C* is determined from the normalisation condition $\omega _- + \omega _+ = 1$. Integrating (48) over *x* implies that the marginal distribution of *s* is a mixture, with weights $\omega _-$ and $\omega _+$, of two truncated Poisson distributions with location parameters $x_-$ and $x_+$ (Fig. 4, right panels). Summing (48) over *s* implies that the marginal distribution of *x* is a mixture, with the same weights, of two Gaussians with means $x_\pm$ and variances $\varepsilon /\varPhi ''(x_\pm )$, which transform to $x_\pm /\varepsilon$ and $1/\varepsilon \varPhi ''(x_\pm )$ in units of molecules $X=x/\varepsilon$ (Fig. 4, left panels).

The second derivative of $\varPhi (x)$, i.e. the first derivative of $\theta =\varPhi '(x)$, is equal at the fixed points to the second component of the nontrivial eigenvector (35) of the Hamiltonian system linearisation (cf. Fig. 1, central panels). Away from the fixed points, the derivative of $\theta =\varPhi '(x)$ can be evaluated by substituting into the right-hand side of (16).

In the chosen example $\varPhi (x_-) > \varPhi (x_+)$, i.e. the right well of the double-well potential is deeper than the left well. Equation (49) then implies that $(\omega _-,\omega _+) \rightarrow (0,1)$ as $\varepsilon \rightarrow 0$. However, the two potential wells are finely balanced, so that the weights are comparable for a range of $\varepsilon \ll 1$, and the two Poissons/Gaussians that constitute the steady-state distribution (48) both contribute.

Figures 3 and 4 demonstrate that the WKB-based Gaussian/Poisson singleton and mixture approximations are in a close agreement with numerical solutions to the chemical master equation; the latter are calculated as follows. First, the CME (4) is truncated to a finite number of equations. Following the approach illustrated e.g. by Borri et al. (2016), we truncate the master equation to a finite lattice $\{0,1,...,s_{\max }\}\times \{0,1,...,X_{\max }\}$, and calculate the (unique) normalised steady-state solution; this amounts to finding a nullvector of a sparse square matrix of large order $({s_\text {max}}+1)(X_{\max }+1)$. The upper bound for the active protein is set to $s_{\max }=20$, while the upper bound $X_{\max }$ for the inactive protein is set to $X_{\max }=4\lceil x_+/\varepsilon \rceil$, where $x_+$ is the uppermost steady state of the rate equation (31). The truncated solution is expected to be a close approximation to the original one because sample trials produced by the stochastic simulation algorithm (Gillespie 1976) (almost) never exceed the upper bound.

## Discussion

We formulated and investigated a stochastic model for the production of a protein with delayed positive feedback. In the model, the protein is produced in bursts of multiple molecule copies. Newly produced protein molecules are inactive, and become activated by passing through a single activation step; biologically, the step can represent chemical modification, compartmental transport, or other scenarios. Active protein molecules regulate the frequency of bursty production of inactive protein. Such feedback can biologically be realised through transcriptional regulation.

The model incorporates an upper bound ${s_\text {max}}$ on the number of active protein. If ${s_\text {max}}$ active protein are already present, a new activation is allowed to occur, but is immediately followed by the removal of the activated molecule; consequently, the number of active protein molecules never exceeds ${s_\text {max}}$. Thanks to the introduction of the upper bound, a number of crucial steps in the mathematical analysis involve finite, rather than infinite, calculation (e.g. the averaging (30) or the matrix (12)). Without an explicit upper bound in the model, each of these calculations would require a numerical truncation; the explicit inclusion of the upper bound in the model guarantees a consistent use of truncation throughout the entire analysis. In the presented numerical examples, we choose ${s_\text {max}}$ large enough in order that the results be close to those expected without an upper bound.

We focused on examining the model behaviour in the $\varepsilon \ll 1$ regime of slow activation. The regime is characterised by an *O*(1)-slow activation rate and $O(1/\varepsilon )$-fast production and decay rates. Consequently, the inactive protein is present at $O(1/\varepsilon )$-large copy numbers and fluctuates slowly on *O*(1)-long timescales, whereas the active protein is present at *O*(1)-low copy numbers and fluctuates fast on $O(\varepsilon )$-short timescales. Neglecting the slow fluctuations in the inactive protein, we found that the active protein obeys a one-dimensional birth–death process which equilibrates to a (truncated) Poisson quasi-steady-state (QSS) distribution. On the slow timescale, the inactive protein is produced with a self-dependent rate that is obtained by averaging the instantaneous production rate with respect to the QSS distribution of the active protein. Depending on whether this effective feedback response function has a single or multiple fixed points, the limiting deterministic dynamics of the inactive protein is monostable or bistable.

Bistability occurs if the effective feedback response function is sufficiently sigmoid. As a result of averaging by the noisy active protein, the effective response function smooths out, or “mollifies”, any sharp features of the instantaneous response function. The requirement that the mollified function be sigmoid implies that the original function must be yet steeper. For simplicity, we used an (infinitely steep) step function in the examples of this paper. Biologically, highly sigmoid feedback responses can be maintained through cooperative binding of the protein to the regulatory DNA sequences.

If the model operates in the slow-activation regime, and the limiting deterministic rate equation is monostable, then the steady-state distribution of the inactive (active) protein is nearly Gaussian (Poisson); the location of the Gaussian/Poisson mode is dictated by the unique fixed point of the rate equation. If the rate equation is bistable, the distribution of the inactive protein is approximated by a mixture of two small-noise Gaussians, and that of the active protein by a mixture of two (moderate-noise) Poissons; the locations of the Gaussian/Poissonian modes are dictated by the fixed points of the rate equation. In order to obtain asymptotic approximations of the weights of the two modes, one needs to consult (and calculate) a WKB solution to the master equation; doing so was the concern of the bulk of the mathematical analysis presented in this paper. The approximate solution closely agrees with a numerical solution to the master equation.

The principal step in the calculation of the asymptotic WKB solution is the determination of the WKB potential. The derivative of the potential is formed by the nontrivial heteroclinic connections between the steady states of a Hamiltonian system (Fig. 1, central panels, solid red curve). The trivial heteroclinic connections that lie on the *x*-axis satisfy the limiting deterministic rate equation (Fig. 1, central panels, dashed red line). The potential derivative and the deterministic rate have opposite signs: the potential has local minima/maxima where the rate equations has stable/unstable fixed points; in other words, the WKB potential is the deterministic rate equation’s Lyapunov function.

Our asymptotic analysis stands on the shoulders of previous analyses (see Introduction for a limited review), and one in particular: Newby and Chapman (2014) study a stochastic gene expression model which is based on different biological assumptions than ours; the commonality is that it features two components with a similar pattern of time and abundance scales. The model of Newby and Chapman (2014) consists of an “internal” finite-state Markov chain (representing promoter states) coupled with an “external” birth and death process (representing protein). The coupling of the two components is in the dependence of transition rates for either component on the current state of both. The deterministic limit in protein dynamics is obtained by reducing both the internal and the external noise. The internal noise is reduced by speeding up the promoter transitions; the external noise is reduced by increasing the protein abundance. If both noise sources are reduced proportionally to each other (and to a small parameter $\varepsilon$), the same configuration of time and abundance scales is achieved as in our model in the slow-activation regime: the promoter state fluctuates at *O*(1) numbers on an $O(\varepsilon )$ timescale and the protein at $O(1/\varepsilon )$ numbers on an *O*(1) timescale. Like we did here for our model, Newby and Chapman (2014) used the WKB method to describe the large-time behaviour of their model in the $\varepsilon \ll 1$ regime. There are some similarities, as well as differences, between the two models as well as in the methodologies of this paper and those of Newby and Chapman (2014). Our model features bursting and a stoichiometric connection between the two species that the model of Newby and Chapman (2014) does not. The WKB potential is determined, in both studies, from the condition that a matrix, here (12), be singular. Our matrix is large and sparse, whereas that of Newby and Chapman (2014) is dense and typically small (few gene states). Methodologically, we define the Hamiltonian as the principal eigenvalue (one with the largest real part), where Newby and Chapman (2014) use the determinant, of the matrix. The method of determining the prefactor (Sect. 5) from the higher-order terms of the master equation is the same as used by Newby and Chapman (2014).

In future work, it will be interesting to look beyond the steady state and quantify the transition rates between the modes $x_-$ and $x_+$ of the mixture distributions identified in the present paper. It is expected that these rates are proportional to $\exp (-(\varPhi (x_0)-\varPhi (x_\pm ))/\varepsilon )$, i.e. exponentially small as $\varepsilon \rightarrow 0$. The proportionality constant will be determined by matching the WKB solution to a solution of a Fokker–Planck equation in the neighbourhood of the unstable fixed point $x_0$ (Hinch and Chapman 2005; Bressloff 2014). We also expect that the current framework can be extended to more general distributed delays. Any non-negative distribution can be arbitrarily closely approximated by a phase-type distribution (Lagershausen 2012). Large deviations driven by a phase-type delay composed of *m* slow memoryless steps will be characterised by a Hamiltonian system with *m* degrees of freedom. On the other hand, a fixed delay does not buffer bursts and is conjectured to generate super-Poissonian distributions at the active protein stage. We expect that the current framework can also be extended to account for multiple protein species. Specifically, in case of a co-repressive toggle switch, we imagine that the relative stabilities of its fixed points can be modulated by the relative lengths of the two delays.

In summary, we performed a detailed analysis of the steady-state distribution for an autoregulating protein with a large one-step production delay. While both monostable and bistable feedbacks can exhibit bimodality at the single-cell level without time delay (Singh 2012; Bokes and Singh 2019), the current results imply that with the inclusion of large delays they will generate qualitatively different distributions. Our analysis thus provides a novel method to probe the structure of positive genetic feedback circuits.

## Appendix: Linearisation of Hamiltonian system

Here we derive expression (37) for the second $\theta$-derivative of the Hamiltonian (18). By doing so, we complete the linearisation analysis of the Hamiltonian system (14).

Differentiating $\varvec{A}\varvec{v} = H \varvec{v}$ with respect to $\theta$ twice yields

$$\begin{aligned} \frac{\partial ^{2}\varvec{A}}{\partial \theta ^{2}} \varvec{v} +2\frac{\partial \varvec{A}}{\partial \theta } \frac{\partial \varvec{v}}{\partial \theta } +\varvec{A} \frac{\partial ^{2}\varvec{v}}{\partial \theta ^{2}} = \frac{\partial ^{2}H}{\partial \theta ^{2}} \varvec{v} + 2 \frac{\partial H}{\partial \theta } \frac{\partial \varvec{v}}{\partial \theta } + H \frac{\partial ^{2}\varvec{v}}{\partial \theta ^{2}}. \end{aligned}$$

At a saddle with coordinates $x=x_*\in \{x_-,x_0,x_+\}$ and $\theta =0$, we have $H (x_*,0) = \frac{\partial H}{\partial \theta } (x_*,0) =0$ and $\varvec{v} (x_*,0) =\varvec{\rho } (x_*)$, so that

$$\begin{aligned} \frac{\partial ^{2} H}{\partial \theta ^{2}} (x_*,0) \varvec{\rho } (x_*)&= \frac{\partial ^{2}\varvec{A}}{\partial \theta ^{2}} (x_*,0) \varvec{\rho } (x_*) +2\frac{\partial \varvec{A}}{\partial \theta } (x_*,0) \frac{\partial \varvec{v}}{\partial \theta } (x_*,0)\\&\quad +\varvec{A} (x_*,0) \frac{\partial ^{2}\varvec{v}}{\partial \theta ^{2}} (x_*,0). \end{aligned}$$

We multiply the above equation by $\varvec{u}^\intercal (x_*,0) = \varvec{1}^\intercal$ from the left; noting that $\varvec{1}^\intercal \varvec{\rho }(x_*)=1$ and $\varvec{1}^\intercal \varvec{A}(x_*,0)=\varvec{0}^\intercal$, we obtain

50 $$\begin{aligned} \frac{\partial ^{2}H}{\partial \theta ^{2}} (x_*,0) = \varvec{1}^\intercal \frac{\partial ^{2}\varvec{A}}{\partial \theta ^{2}} (x_*,0)\varvec{\rho }(x_*) + 2 \varvec{1}^\intercal \frac{\partial \varvec{A}}{\partial \theta } (x_*,0) \frac{\partial \varvec{v}}{\partial \theta } (x_*,0). \end{aligned}$$

The second derivative of $\varvec{A}(x,\theta )$ is the matrix representation of the second derivative

$$\begin{aligned} \frac{\partial ^{2}{\mathcal {A}}}{\partial \theta ^{2}} (x,\theta ) v_s = \text {e}^{-\theta } x {\mathcal {R}} v_s + M''(\theta ) f_s v_s \end{aligned}$$

of the operator ${\mathcal {A}}(x,\theta )$ (11). Therefore, the first term on the right-hand side of (50) satisfies

51 $$\begin{aligned} \varvec{1}^\intercal \frac{\partial ^{2}\varvec{A}}{\partial \theta ^{2}} (x_*,0) \varvec{\rho } (x_*)&= \sum _{s=0}^{{s_\text {max}}} \frac{\partial ^{2}{\mathcal {A}}}{\partial \theta ^{2}} (x_*,0) \rho _s (x_*)\nonumber \\&= x_* \sum _{s=0}^{{s_\text {max}}} {\mathcal {R}} \rho _s(x_*) + M''(0) \sum _{s=0}^{s_\text {max}}f_s \rho _s(x_*)\nonumber \\&= x_* + \langle B^2 \rangle {\bar{f}}(x_*) = x_*\left( 1 + \frac{\langle B^2 \rangle }{\langle B \rangle }\right) , \end{aligned}$$

in which we utilised the fact that $x_*$ is a fixed point of $\langle B \rangle {\bar{f}}(x)$.

The second term on the right-hand side of (50) involves the derivative of the principal eigenvector with respect to $\theta$. Differentiating $\varvec{A} \varvec{v} = H \varvec{v}$ with respect to $\theta$ and inserting $x=x_*$ and $\theta =0$ yields

52 $$\begin{aligned} \varvec{A} (x_*,0) \frac{\partial \varvec{v}}{\partial \theta } (x_*,0) = - \frac{\partial \varvec{A}}{\partial \theta } (x_*,0) \varvec{\rho } (x_*). \end{aligned}$$

Solving the inhomogeneous linear algebraic system (52) yields

$$\begin{aligned} \frac{\partial \varvec{v}}{\partial \theta } (x_*,0) = \tilde{\varvec{v}} + c \varvec{\rho }(x_*), \end{aligned}$$

where $c \varvec{\rho }(x_*)$ is a representant of the (right) kernel of $\varvec{A}(x_*,0)$ and

$$\begin{aligned} \tilde{\varvec{v}} = - [\varvec{A} (x_*,0)]^+ \frac{\partial \varvec{A}}{\partial \theta } (x_*,0) \varvec{\rho } (x_*) \end{aligned}$$

is a least-squares solution to (52), with $\varvec{A}^+$ denoting the pseudo-inverse of $\varvec{A}$.

The second term in (50) therefore satisfies

53 $$\begin{aligned} \varvec{1}^\intercal \frac{\partial \varvec{A}}{\partial \theta } (x_*,0) \frac{\partial \varvec{v}}{\partial \theta } (x_*,0)&= \varvec{1}^\intercal \frac{\partial \varvec{A}}{\partial \theta } (x_*,0) \tilde{\varvec{v}} + c \varvec{1}^\intercal \frac{\partial \varvec{A}}{\partial \theta } (x_*,0) \varvec{\rho } (x_*)\nonumber \\&= \varvec{1}^\intercal \frac{\partial \varvec{A}}{\partial \theta } (x_*,0) \tilde{\varvec{v}} + c \frac{\partial H}{\partial \theta } (x_*,0)\nonumber \\&= \varvec{1}^\intercal \frac{\partial \varvec{A}}{\partial \theta } (x_*,0) \tilde{\varvec{v}}. \end{aligned}$$

Inserting (51) and (53) into (50) recovers (37).
